# Evaluation of the Enzyme Inhibition, Antioxidant, and Antimicrobial Activities of Apricots, Plums, and Their Hybrid Fruits

**DOI:** 10.3390/plants13202936

**Published:** 2024-10-20

**Authors:** Dasha Mihaylova, Ivelina Desseva, Yulian Tumbarski, Aneta Popova, Svetla Pandova, Anna Lante

**Affiliations:** 1 Department of Biotechnology, University of Food Technologies, 4002 Plovdiv, Bulgaria; 2 Department of Analytical Chemistry and Physical Chemistry, University of Food Technologies, 4002 Plovdiv, Bulgaria; 3 Department of Microbiology, University of Food Technologies, 4002 Plovdiv, Bulgaria; 4 Department of Biochemistry and Nutrition, University of Food Technologies, 4002 Plovdiv, Bulgaria; 5 Department of Breeding and Genetic Resources, Fruit Growing Institute, Agricultural Academy, 4000 Plovdiv, Bulgaria; 6 Department of Agronomy, Food, Natural Resources, Animals, and Environment—DAFNAE, University of Padova, 35020 Legnaro, Italy

**Keywords:** biological activity, *Prunus* spp., free phenolic compounds, bound phenolic compounds, bacteria, fungi, glucosidase, acetylcholinesterase, lipase

## Abstract

The current study focuses on evaluating the enzyme inhibition (acetylcholinesterase, α-amylase, α-glucosidase, pancreatin lipase), antioxidant, and antimicrobial activities of the “Modesto” apricot, “Stanley” plum, and their hybrid the “Stendesto” plum–apricot. The “Stendesto” is the only successful plum–apricot hybrid in Bulgaria. A spectrophotometric approach was used to evaluate the antioxidant activity following four complementary assays (DPPH, ABTS, FRAP, and CUPRAC). The “Stendesto” plum–apricot revealed its enhanced antioxidant potential compared to its parental lines. Apart from the free phenolics extraction, two other techniques (alkaline and acid hydrolysis) were applied to reveal the biological potential of the studied fruit. Not only free but also bound phenolic extracts were able to inhibit α-glucosidase and acetylcholinesterase, while none of the extracts inhibited lipase or α-amylase. None of the apricot extracts had antimicrobial activity, while the other fruit had limited antimicrobial activity. The proposed results undoubtedly reveal that hybrid fruits possess enhanced biological activity compared to their parents. This is a first comprehensive evaluation of hybrid fruits with reference to parental lines. This makes them an interesting research topic that should be better explored.

## 1. Introduction

Plants are the most important source of bioactive molecules which greatly contribute to the functional role of nutrition [1]. Phenolic compounds are often identified in different plant bases, incl. fruit, which makes them some of the most recognized molecules with biological activity [2]. Polyphenols can influence the taste and appearance of fruit [3]. Flavonoids are revealed as major sources of polyphenols in the human diet [4]. Anthocyanins are not only recognized as powerful natural pigments but also have antioxidant, anti-inflammatory, antidiabetic, anticancer and cardiovascular properties [5]. Phenolic acids, flavonoids, and anthocyanins, among others, are extracted using different techniques in order to prove their biological values [6]. Both free and bound phenolic compounds are recovered from plant matrices but the current research emphasis falls on the bound ones and their properties [7]. Free phenolics are easily extractable and absorbed in the small intestine, while bound phenolics require specificity based on their solubility or insolubility and are absorbed after reaching the colon [8]. The difference between soluble-bound phenolics and insoluble ones is in the links they form. Soluble-bound phenolics are linked to one or more sugar units (through a hydroxyl group or carbon–carbon linkages) while insoluble-bound phenolics covalently bind to cell wall cellulose, hemicellulose, lignin, pectin, and rod-shaped structural proteins forming stable complexes [8]. Phenolic compounds in general are continuously reported for their antioxidant, antiviral, cardioprotective, cancer, and metabolic management activities [9]. Polyphenols also show antibacterial activity against a large number of Gram-positive and Gram-negative bacteria [10]. This is of particular importance with the rising resistance of bacteria to conventional drugs [11]. Furthermore, polyphenols are positively associated with the management of gut microbiota health [12].

Some of the major societal challenges include hyperlipidemia, hyperglycemia, cardiovascular disease, inflammation, and cancer [13,14,15]. Their manifestations are linked to oxidative stress, thus identifying low-cost, natural, and efficient compounds with antioxidant and other biological activities, and have set an ongoing path for researchers. Different fruit from the *Prunus* spp., incl. apricots and plums, are known for their antidiabetic and cardioprotective activities [16]. Apricots contain carotenoids and ascorbic acid as well as polyphenols [17]. Plums are known for the presence of epicatechin and neochlorogenic acid as free phenolics, and catechin and epicatechin as the main bound phenolics [16].

Currently, many breeding programs focus on hybridization aiming mainly at eliminating common diseases or targeting an elevated sugar content [18]. Moreover, the modern consumer is constantly demanding new products with enhanced properties. A current review suggests that hybridization may produce fruits that are a source of antioxidant and bioactive compounds and act as human health managers [19]. Plum–apricot hybrids are the result of the successful crossing between highly desirable fruits like the apricot and the plum. Plum–apricot hybridization may result in pluots, apriums, or plumcots [20]. The “Stendesto” is the only Bulgarian plum–apricot, mostly visually resembling a plum. Plum–apricot hybrids are reported to inherit all of their parent’s metabolites [21] but there is not enough research on the topic.

In view of the above, it is of interest to obtain phenolic compounds from hybrid fruits and set a reference to their parental lines bearing in mind their biological activity. In addition, a major gap in the research of hybrid fruit exists, which makes this a pilot study on the topic and a stepping stone for future exploration.

## 2. Results and Discussion

Polyphenols are widely recognized and are some of the most prominent antioxidants with proven effectiveness [22]. Polyphenols are the principal antioxidant compounds that can counteract free radicals in the body. Anthocyanins are the color pigments that mainly contribute to the blue, purple, and red colors of fruits and vegetables. They have antioxidant [23], anti-inflammatory [24], antidiabetic [25], anticancer [26] and neuro- and cardioprotective activities [27]. Table 1 summarizes data on the content of polyphenols, flavonoids, and anthocyanins in the studied fruits. In order to thoroughly evaluate the samples, the extraction of both free and bound phenolic compounds was carried out. The extraction of bound phenolics was carried out by two widely used approaches differing in conditions, namely alkaline and acid hydrolysis, in order to find the one more suitable for the investigated fruits.

The plum–apricot hybrid revealed its highest results for total phenolic content, total flavonoid content, and total monomeric anthocyanins in the free phenolic extract. However, in the bound phenolic extracts, the alkaline hydrolysis resulted in higher values compared to the acid one. The acid hydrolysis produced its highest values in the TPCs of the “Modesto” apricot. These findings suggest that, indeed, hybridization in fruits results in enhanced biological properties. The established TPCs values for plums vary widely in published papers. The values measured for TFs vary from 3.12 to 20.63 mg QE/g [28], which once again proves that not only the variety and growing conditions but also the type of extraction have an influence on the extraction of biologically active compounds. Liaudanskas et al. [29] reported that flavonols accounted for 25.8% of the TPC of plums from the “Stanley” variety. Vlaic et al. [30] found values of 5.56–261.93 mg CE 100 g^−1^ (TMAs) for plums from the “Stanley” variety. The data available in the literature correspond to the values obtained in the present study for total phenolic content, total flavonoid content, and total monomeric anthocyanins of “Stanley” plum fruits.

Similar to plum fruits, a wide range of data on TPCs and TFs values are reported when analyzing apricots, e.g., for ultrasonic extraction, the TPC was about 165.49 mg GAE/100 g DM [31]. Corresponding to the current results, Dulf et al. [32] also report that the free phenolic extract had higher results compared to the bound one. Other authors found values from 4233.70 to 8180.49 mg of GAE/100 g dry matter for the TPCs of different apricot cultivars grown in regions of Turkey, Pakistan, and Chile [33,34]. Contrary to the current results, Tareen et al. [35] reported amounts of flavonoids and anthocyanins in apricot pomace.

The “Stendesto” hybrid is characterized by having the largest amount of free phenolic compounds as well as total flavonoids and total monomeric anthocyanins. The present data are the first for hybrid fruits, which will add to the existing scientific base and enable future comparisons. A greater similarity is observed between plum–apricot and plum than between plum–apricot and apricot.

Table 2 presents the results of the evaluation of the antioxidant potential of the studied fruits.

The antioxidant potential of the fruit extracts of free phenolic compounds showed higher values for all studied fruits compared to the extracts (acid and alkaline) of bound ones. The fruits of the “Stendesto” hybrid show data more similar to those of the “Stanley” plum than the ”Modesto” apricot. The “Stendesto” hybrid exhibited the highest values for antioxidant potential in almost all applied assays. Its antioxidant potential was nearly fivefold greater than that of the apricot. This may suggest that hybrid fruits have enhanced biological values compared to their parental lines.

There are data in literature on the antioxidant activity of plums from the ”Stanley” variety, where authors reported data on juices, peels, and fruits. However, no data were found for extracts of free and bound phenolic compounds, nor was an assessment made using more than one method measuring the antioxidant potential. The present results represent new complex data that will add to what is available in the literature to date. Miletić et al. [36] investigated the antioxidant potential of plums from the ”Stanley” variety and found no trends during fruit development. There are also data on the antioxidant potential of apricots, but the “Modesto” variety has not been studied. However, high FRAP data are reported for other cultivars [37].

Ozzengin et al. [38] reported that the DPPH assay revealed the highest values in plums from the Karaca and Üryani varieties, which strongly suggests that each variety of the same fruit has a different biological value.

The acetylcholinesterase-inhibitory potential of the samples was also evaluated. For this purpose, the extracts were studied by an in vitro approach and the results are reflected in Table 3.

Acetylcholinesterase (AChE) has an important part in neurodegenerative diseases’ pathogenesis by guiding the inflammatory response, apoptosis, oxidative stress, and aggregation of pathological proteins. New compounds that can inhibit the beginning of neurodegenerative diseases and slow their evolution are constantly being sought [39]. AChE potential was detected only in the fruits of the plum–apricot hybrid and plum. Aluko [40] reported that the aqueous extracts of plums were effective with an IC_50_ value of 16.75 mg/mL. These data are comparable to those obtained in the present study. However, this activity is low compared to IC_50_ values of the extracts from other fruits, which have been shown to be <1 mg/mL.

There are reports about the AChE activity of *Prunus* spp. leaves where apricot leaves were more active compared to plum ones [41]. Additionally, apricot kernels were also found to be effective AChE inhibitors [42].

In vitro analyses were also carried out regarding the inhibitory potential against the digestive enzymes of the extracts. The results are summarized in Table 4.

Activity was found in the plum and plum–apricot extracts, while no activity was present in the apricot ones. The lack of activity in apricots may be due to the fact that total monomeric anthocyanins and total flavonoids were not detected there especially in the bound form (Table 1). However, Wojdyło and Nowicka [43] reported an inhibitory activity, but for apricot leaves. The ”Stanley” plum showed better potential compared to the hybrid fruit. Other authors found that the fruits of different varieties of *P. domestica* showed inhibitory activity ((IC_50_) mg/mL) against α-amylase (2.63–61.53), α-glucosidase (0.19–24.07), and pancreatin lipase (0.50–8.20) [44]. The same authors, however, also report that not every variety of plum fruits studied exhibited activity. Human pancreatic α-amylase and intestinal α-glucosidase are accountable for the hydrolysis of carbohydrates into digestible simple sugars. Inhibition of these enzymes lowers blood sugar levels by limiting the breakdown of polysaccharides to glucose [45]. Extracts and isolated compounds from plant sources are believed to be able to inhibit α-amylase, and flavonoids exhibit the greatest inhibition potential related to the number of hydroxyl groups in their molecules [46]. Pancreatic lipase is an important enzyme accountable for the hydrolysis of dietary fats to monoacylglycerols and free fatty acids. This helps decrease overweight and obesity in diabetic patients by significantly controlling the inhibitory effects of fat absorbed into the bloodstream [47]. Additionally, the enzyme is recommended as a means of reducing weight. Fruits can effectively inhibit pancreatic lipase, but this ability strongly depends on the variety.

It has been suggested that phenolic compounds (i.e., flavonoids and phenolic acids) may demonstrate antimicrobial properties [48]. For this reason, the antimicrobial activity of the extracts of the studied fruits was estimated (Table 5) as a marker for their biological potential. No particularly high inhibition was found to estimate the minimum inhibitory concentration (MIC). Still, the antibacterial potential is important for fruits to repair injuries and/or have an extended shelf life [49]. Regarding both Gram-positive and Gram-negative bacteria and yeasts, the associated phenolic fractions had almost no activity. The extracts were also tested against *Candida albicans* NBIMCC 74; *Saccharomyces cerevisiae* ATCC 9763; *Aspergillus niger* ATCC 1015; *Aspergillus flavus*; *Penicillium chrysogenum*; and *Fusarium moniliforme* ATCC 38932. No activity was recorded and the same are not present in the report table. The “Modesto” apricot showed no antimicrobial activity at all, and it is not presented in the following table.

**Figure 1:**
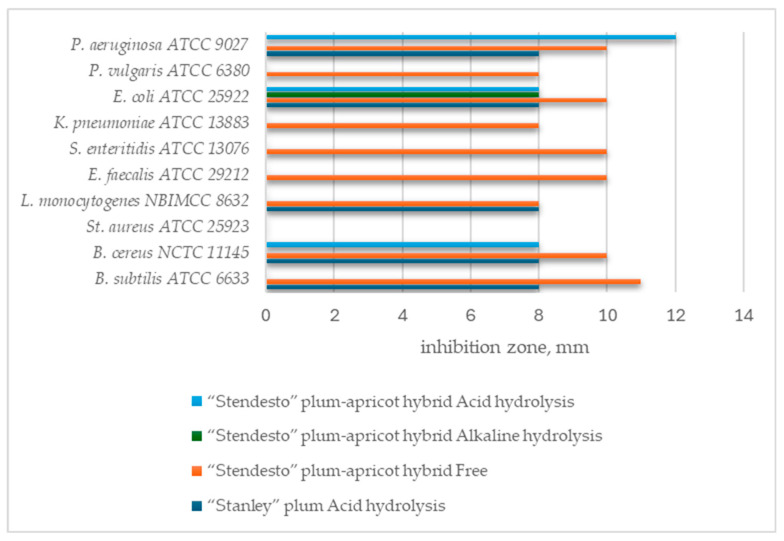
Inhibitory effect of extracts of plum and plum–apricot fruit on bacteria, yeasts, and fungi.

The inhibitory effect of the related phenols was more pronounced against *Bacillus subtilis* ATCC 6633, *Bacillus cereus* NCTC 11145, *E. coli* ATCC 25922, and *P. aeruginosa* ATCC 9027 (Figure 1).

Other authors reported comparable results for plum extracts (the variety was not mentioned) regarding *E. coli* and *Listeria monocytogenes* [50]. This is also consistent with the present findings as well as their relationship with polyphenol content and their antioxidant activity. The obtained data on the inhibitory effect of plum–apricot hybrid fruit extracts can be considered new data due to the lack of available information in the literature for comparison. Other authors reported that apricot extracts (seeds and pulp) had a reaction against *E. coli*, *L. monocytogenes*, *P. aeruginosa PA14*, *S. typhimurium*, and *S. aureus* [51]. Molecular weight, polarity, and side groups determine the specific inhibitory effect of each phenolic compound [52]. Phenolic acids (ferulic acid and p-coumaric acid), alcohols (e.g., catechol and vanillyl alcohol), and aldehydes (vanillin and syringaldehyde) are thought to be the most effective inhibitors of microbial growth [53]. Additionally, organic acids may be accountable for their activity against Gram-(+) and Gram-(−) bacteria, due not only to their abundance and different biochemical nature, but also to their capability of reducing the pH [54].

## 3. Materials and Methods

### 3.1. Fruit Samples

Apricot, plum–apricot, and plum fruits were harvested on three dates according to their specific ripeness from the fields of the Fruit Growing Institute, Plovdiv, Bulgaria. A total of sixty fruits per variety, with an extra twenty in case of need, were transported in pulp trays in an air-conditioned vehicle to the laboratories of the University of food technologies, Plovdiv, Bulgaria, where the fruits were randomly placed in new trays in order to minimize the differences in fruit quality. Consequently, they were washed, sliced with a ceramic knife, and frozen in vacuum-sealed bags. After 24 h in the freezer, the fruits were subjected to lyophilization in a vacuum freeze dryer (BK-FD12S, Biobase, Jinan, Shandong, China) under the pressure of 3.5 MPa at −55 °C. The resulting samples were then powdered with a Tefal GT110838 grinder (Rumilly, France) at 180 W for 30 s and kept in air-tight containers prior to extraction.

### 3.2. Extraction of Free and Bound Phenolic Compounds

#### 3.2.1. Extraction of Free Phenolic Compounds

A threefold extraction of free phenolic compounds [55] of each fruit sample was performed by mixing 0.5 g of sample with 10 mL 80% (80:16, *v*/*v*) ethanol. The mixture was ultrasonically (UST 5.7150 Siel, Gabrovo, Bulgaria) extracted at 70 °C for 30 min and centrifuged at 10,000× *g* for 20 min. The resulting phenolic extracts, after combining, were filtered using filter paper (Whatman No. 1) and evaporated until dry (RV 10, Ika, Staufen, Germany). The final volume of the extracts was adjusted by adding 10 mL of 80% methanol (80:20, *v*/*v*) and stored at −20 °C until further analysis.

#### 3.2.2. Extraction of Bound Phenolic Compounds

The bound phenolic compounds were extracted by two protocols—the alkaline hydrolysis method and the acid hydrolysis method, respectively. The alkaline extraction procedure was conducted according to the method described by Ding et al. [56] with modifications [55], and the acid extraction procedure was carried out as previously reported by Mihaylova et al. [55]. Both dried bound extracts were reconstituted each in 10 mL 80% HPLC-grade methanol (80:20, *v*/*v*) and stored unilluminated at −20 °C until analysis.

### 3.3. Evaluation of the Total Phenolic Content (TPCs)

The TPCs was evaluated following a modified method of Kujala et al. [57] where a 0.1 mL sample was mixed with 0.5 mL Folin–Ciocalteu reagent followed by 0.4 mL 7.5% Na_2_CO_3_. The mixture was vortexed and incubated for 5 min at 50 °C. After that, the absorbance was measured at 765 nm. The result is expressed as mg gallic acid equivalents (GAEs) per g dry weight (mg GAE/g dw).

### 3.4. Evaluation of Total Flavonoid Content (TFCs)

The method of Kivrak et al. [58] was applied to evaluate the total flavonoid content. An aliquot of 0.5 mL of the sample was mixed with 0.1 mL of 10% Al(NO_3_)_3_, 0.1 mL of 1 M CH_3_COOK, and 3.8 mL of ethanol. The mixture was incubated at room temperature for 40 min and the absorbance was measured at 415 nm. Quercetin (QE) was used as a standard, and the results are expressed as μg quercetin equivalents (QEs)/g dw.

### 3.5. Determination of Total Monomeric Anthocyanins (TMAs)

The TMAs content was defined using the pH differential method [59] as described by Mihaylova et al. [60]. Results are expressed as µg cyanidin-3-glucoside (C3GE)/g dw.

### 3.6. Evaluation of Antioxidant Activity Potential (AOA)

The antioxidant activity potential assessment was performed using four widely used and reliable in vitro methods, namely the DPPH^•^ Radical Scavenging Assay (according to the method of Brand-Williams et al. [61] with slight modifications described by Mihaylova et al. [62]), ABTS^•+^ Radical Scavenging Assay (Re et al. [63]), Ferric-Reducing Antioxidant Power (FRAP) Assay (Benzie and Strain [64]), and Cupric Ion-Reducing Antioxidant Capacity (CUPRAC) Assay (Apak et al. [65]).

### 3.7. Evaluation of Enzyme-Inhibitory Activities

#### 3.7.1. α-Amylase (AM)-Inhibitory Assay

Each extract was mixed with an enzyme solution (1:1, *v*/*v*) to achieve a final concentration of 1 U/mL α-amylase and left for 15 min at 23 °C. The remaining α-amylase activity was completed exactly as descried by the Sigma Aldrich method [66]. Enzyme without inhibitors was used as a negative control. The absorbance was measured at 540 nm and results are expressed as the concentration of the extract (IC_50_) in mg/mL that inhibited 50% of α-amylase.

#### 3.7.2. α-Glucosidase (AG)-Inhibitory Assay

The reaction mixture containing 10 µL of the extract (a minimum of five extract concentrations were tested in order to calculate the IC_50_) and 30 µL of α-glucosidase (0.1 U/mL, G5003-100UN, Sigma-Aldrich, Merck, Darmstadt, Germany) was incubated for 15 min at 37 °C in a microplate reader (SPECTROstar Nano Microplate Reader, BMG LABTECH, Ortenberg, Germany). Next, 25 µL of 1 mM 4-nitrophenyl-α-D-glucopyranoside (N 1377, Sigma-Aldrich, Merck, Darmstadt, Germany) was added. The reaction mixture was then shaken and incubated at 37 °C for 10 min. The reaction was terminated by adding 60 µL of 0.2 M Na_2_CO_3_ solution. Blanks were prepared by adding the extract after the termination of the reaction. Enzyme without inhibitor was used as a negative control. The absorbance (405 nm) was measured using a microplate reader and results are expressed as a concentration of the extract (IC_50_) in mg/mL that inhibited 50% of α-glucosidase [67].

#### 3.7.3. Pancreatic Lipase-Inhibitory Assay

The in vitro pancreatic lipase-inhibitory activity was determined as described by Saifuddin et al. [68] and Dobrev et al. [69] with slight modifications. The results are expressed as concentration of the extract (IC_50_) in mg/mL that inhibited 50% of pancreatic lipase.

#### 3.7.4. Acetylcholineesterase (AChE)-Inhibitory Assay

The experimental conditions of the in vitro AChE-inhibitory assay were based on the method described by Lobbens et al. [70] with some modifications as described by Mihaylova et al. [55]. The results are expressed as a concentration of the extract (IC_50_) in mg/mL that inhibited 50% of acetylcholinesterase.

### 3.8. Antimicrobial Activity

Four Gram-positive bacteria (*Bacillus subtilis* ATCC 6633, *Staphylococcus aureus* ATCC 25923, *Listeria monocytogenes* NBIMCC 8632, and *Enterococcus faecalis* ATCC 19433), four Gram-negative bacteria (*Salmonella enteritidis* ATCC 13076, *Escherichia coli* ATCC 8739, *Proteus vulgaris* ATCC 6380, and *Pseudomonas aeruginosa* ATCC 9027), two yeasts (*Candida albicans* NBIMCC 74 and *Saccharomyces cerevisiae* ATCC 9763) and six fungi (*Aspergillus niger* ATCC 1015, *Aspergillus flavus*, *Penicillium* sp., *Rhizopus* sp., *Mucor* sp.-plant isolates, and *Fusarium moniliforme* ATCC 38932) from the collection of the Department of Microbiology at the University of Food Technologies, Plovdiv, Bulgaria, were selected for the antimicrobial activity test. Luria–Bertani agar medium supplemented with glucose (LBG) was prepared as prescribed by the manufacturer (Laboratorios Conda S.A., Madrid, Spain). Malt extract agar (MEA) was prepared as suggested by the manufacturer (HiMedia^®^, Thane, India). The assay was performed exactly as described by Mihaylova et al. [55].

### 3.9. Statistical Analyses

Results are expressed as the mean  ±  SD (threefold). The impact of fruit and extraction type on the TPC, TFC, TMA, and AOA was estimated using a two-factor variance analysis [71]. The Tukey–Kramer post hoc test (α = 0.05) aided in the statistical comparison of the data [71].

## 4. Conclusions

This study presents new information about plum–apricot hybrids and enlarges the data available about plums and apricots of specific varieties. It focuses on evaluating the enzyme inhibition, antioxidant, and antimicrobial activities of the “Modesto” apricot, the “Stanley” plum, and their hybrid the “Stendesto” plum–apricot. Three types of extracts for each fruit were prepared to assess their biological activity. The “Stendesto” plum–apricot revealed its enhanced activities compared to its parental lines. It showed better antioxidant activity, especially considering the ABTS assay. The enzyme-inhibitory potential was rather low in all three studied fruits and non-existent towards lipase and α-amylase. However, the “Stendesto” plum–apricot had limited inhibition on acetylcholinesterase and α-glucosidase. Additionally, the extract of free phenolics of the “Stendesto” plum–apricot had better antimicrobial activity compared not only to the “Stanley” plum (maternal) but also to the “Modesto” apricot (paternal). These results can effectively be used as a reference work and trampoline for future analyses.

## Figures and Tables

**Table 1:** Total phenolic compounds (TPCs) (mgGAE/g dw), total flavonoids (TFs) (μgQE/g dw), and total monomeric anthocyanins (TMAs) (μg cyanidin-3-glucoside (C3GE)/g dw) in studied extracts.

Samples/Assays		TPCs	TFs	TMAs
“Stendesto” plum–apricot hybrid	Free	23.52 ± 0.75 ^a^	5840.7 ± 108.8 ^a^	1502.70 ± 24.10 ^a^
	Alkaline hydrolysis	0.72 ± 0.01 ^d^	76.5 ± 0.9 ^d^	25.61 ± 1.09 ^c^
	Acid hydrolysis	0.40 ± 0.00 ^d^	-	-
“Stanley” plum	Free	19.08 ± 0.58 ^b^	1971.96 ± 13.32 ^c^	1219.96 ± 34.29 ^b^
	Alkaline hydrolysis	0.61 ± 0.01 ^d^	54.04 ± 3.55 ^d^	19.71 ± 1.34 ^c^
	Acid hydrolysis	0.50 ± 0.01 ^d^	25.37 ± 5.54 ^d^	-
“Modesto” apricot	Free	4.45 ± 0.09 ^c^	3277.71 ± 11.79 ^b^	-
	Alkaline hydrolysis	1.01 ± 0.00 ^d^	-	-
	Acid hydrolysis	0.97 ± 0.01 ^d^	-	-

Different letters in the same column indicate statistically significant differences (*p* < 0.05) according to ANOVA and the Tukey test.

**Table 2:** Antioxidant profile of fruit extracts according to four in vitro methods (DPPH, ABTS, FRAP and CUPRAC, µM/g dw).

Sample/Assay		DPPH	ABTS	FRAP	CUPRAC
“Stendesto” plum–apricot hybrid	Free	58.86 ± 1.46 ^a^	340.27 ± 1.54 ^a^	173.77 ± 2.60 ^a^	260.16 ± 4.49 ^a^
	Alkaline hydrolysis	1.79 ± 0.01 ^d^	10.14 ± 1.82 ^d^	4.16 ± 0.08 ^d^	7.52 ± 0.19 ^d^
	Acid hydrolysis	1.03 ± 0.03 ^d^	8.15 ± 0.45 ^d^	1.07 ± 0.02 ^d^	5.07 ± 0.07 ^d^
“Stanley” plum	Free	52.78 ± 0.58 ^b^	239.50 ± 4.90 ^b^	136.57 ± 6.73 ^b^	191.85 ± 1.65 ^b^
	Alkaline hydrolysis	1.517 ± 0.026 ^d^	˂LOD	2.77 ± 0.05 ^d^	5.65 ± 0.34 ^d^
	Acid hydrolysis	1.146 ± 0.036 ^d^	˂LOD	2.81 ± 0.04 ^d^	5.92 ± 0.25 ^d^
“Modesto” apricot	Free	12.98 ± 0.06 ^c^	64.20 ± 0.39 ^c^	33.96 ± 0.16 ^c^	55.31 ± 0.63 ^c^
	Alkaline hydrolysis	0.54 ± 0.06 ^d^	˂LOD	2.98 ± 0.06 ^d^	6.48 ± 0.03 ^d^
	Acid hydrolysis	0.35 ± 0.02 ^d^	˂LOD	2.32 ± 0.08 ^d^	5.40 ± 0.25 ^d^

˂LOD—level of detection; different letters in the same column indicate statistically significant differences (*p* < 0.05) according to ANOVA and the Tukey test.

**Table 3:** Acetylcholinesterase-inhibitory potential of extracts of bound and free phenolic compounds of studied fruits, IC_50_, g/mL.

Sample	Extraction Type	IC_50_, g/mL
“Stendesto” plum–apricot hybrid	Free	0.0219 ± 0.001 ^b^
	Alkaline hydrolysis	-
	Acid hydrolysis	-
“Stanley” plum	Free	0.0547 ± 0.003 ^a^
	Alkaline hydrolysis	0.0584 ± 0.002 ^b^
	Acid hydrolysis	-
“Modesto” apricot	Free	-
	Alkaline hydrolysis	-
	Acid hydrolysis	-

Different letters in the same column indicate statistically significant differences (*p* < 0.05) according to ANOVA and the Tukey test.

**Table 4:** Enzyme-inhibitory potential against digestive enzymes (α-glucosidase, lipase, and α-amylase) of extracts of bound and free phenolic compounds of fruits, IC_50_, g/mL.

Sample/Assay		α-Glucosidase	Lipase	α-Amylase
“Stendesto” plum–apricot hybrid	Free	0.00425 ± 0.001 ^c^	-	-
	Alkaline hydrolysis	0.0416 ± 0.001 ^a^	-	-
	Acid hydrolysis	-	-	-
“Stanley” plum	Free	0.005254 ± 0.0003 ^c^	-	-
	Alkaline hydrolysis	0.029735 ± 0.002 ^b^	-	-
	Acid hydrolysis	-	-	-
“Modesto” apricot	Free	-	-	-
	Alkaline hydrolysis	-	-	-
	Acid hydrolysis	-	-	-

Different letters in the same column indicate statistically significant differences (*p* < 0.05) according to ANOVA and the Tukey test.

**Table 5:** Effect of free and bound phenols from plum and plum–apricot fruit extracts on antimicrobial potential against bacteria, yeasts, and fungi.

Test Microorganisms	Inhibition Zone, mm					
Sample	“Stanley” Plum			“Stendesto” Plum–Apricot Hybrid		
Extraction	Free	Alkaline Hydrolysis	Acid Hydrolysis	Free	Alkaline Hydrolysis	Acid Hydrolysis
*B. subtilis* ATCC 6633	8	-	8	11	-	-
*B. cereus* NCTC 11145	8	-	8	10	-	8
*St. aureus* ATCC 25923	-	-	-	-	-	-
*L. monocytogenes* NBIMCC 8632	-	-	8	8	-	-
*E. faecalis* ATCC 29212	-	-	-	10	-	-
*S. enteritidis* ATCC 13076	-	-	-	10	-	-
*K. pneumoniae* ATCC 13883	-	-	-	8	-	-
*E. coli* ATCC 25922	8	8	8	10	8	8
*P. vulgaris* ATCC 6380	-	-	-	8	-	-
*P. aeruginosa* ATCC 9027	-	-	8	10	-	12

## Data Availability

The data presented in this study are available on request from the corresponding authors.
